# The alternative proteome in neurobiology

**DOI:** 10.3389/fncel.2022.1019680

**Published:** 2022-11-17

**Authors:** Pablo Mohaupt, Xavier Roucou, Constance Delaby, Jérôme Vialaret, Sylvain Lehmann, Christophe Hirtz

**Affiliations:** ^1^LBPC-PPC, Université de Montpellier, IRMB CHU de Montpellier, INM INSERM, Montpellier, France; ^2^Department of Biochemistry and Functional Genomics, Université de Sherbrooke, Sherbrooke, QC, Canada

**Keywords:** mRNA, non-coding RNA (ncRNA), neurobiology, polycistronic, proteome

## Abstract

Translation involves the biosynthesis of a protein sequence following the decoding of the genetic information embedded in a messenger RNA (mRNA). Typically, the eukaryotic mRNA was considered to be inherently monocistronic, but this paradigm is not in agreement with the translational landscape of cells, tissues, and organs. Recent ribosome sequencing (Ribo-seq) and proteomics studies show that, in addition to currently annotated reference proteins (RefProt), other proteins termed alternative proteins (AltProts), and microproteins are encoded in regions of mRNAs thought to be untranslated or in transcripts annotated as non-coding. This experimental evidence expands the repertoire of functional proteins within a cell and potentially provides important information on biological processes. This review explores the hitherto overlooked alternative proteome in neurobiology and considers the role of AltProts in pathological and healthy neuromolecular processes.

## Introduction

The quantitative composition of molecules in living systems is constantly changing (Cohn et al., 1976; Filo et al., 2019; Lehallier et al., 2019). Multiple mechanisms modulate the molecular landscape in response to a changing environment (Stadler et al., 2008; Geyer et al., 2016; Thiemicke et al., 2019). These variations in the molecular landscape are particularly reflected in the proteome and the interactome (Aebersold et al., 2018). Recently, the Human Proteome Organization reported a meritorious human proteome coverage of 90.4% (Adhikari et al., 2020). This coverage is based on the number of proteins with evidence of their existence at the protein level out of the total protein entries in the NeXtProt database. However, 75% of spectra in large-scale mass spectrometry-based proteomics studies are not matched to a specific protein (Griss et al., 2016). Although a fraction of spectra remains unidentified because of unknown post-translational modifications or low signal-to-noise events, another fraction of unmatched spectra likely results from the incompleteness of conventional protein sequence databases.

**FIGURE 1:**
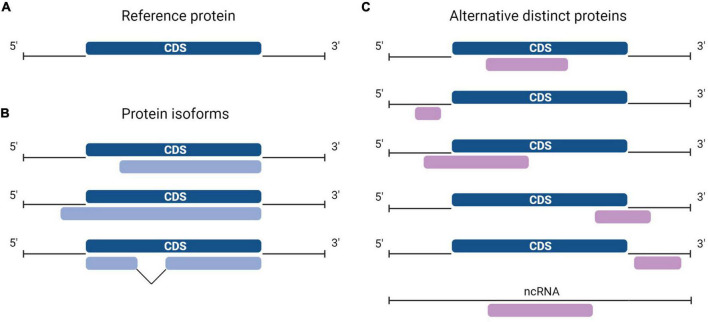
Diversity in the location of protein-coding sequences (CDSs). **(A)** In a typical messenger RNA (mRNA), the canonical protein CDS, or CDS (dark blue) encodes the reference protein (RefProt) and is flanked by the 5′- and 3′- untranslated regions (UTRs). **(B)** Protein isoforms from the RefProt (light blue) may result from translation initiation at upstream or downstream initiation sites, or from the translation of alternatively spliced transcripts. **(C)** In addition to the conventional CDS, an mRNA may carry other CDSs or alternative open reading frames (ORFs) (purple), alternative ORFs (AltORFs) are present in regions of the transcriptome previously annotated as non-coding: 5′- and 3′-UTR, overlapping the CDS out-of-frame, and transcripts annotated as non-coding RNA (ncRNA).

Protein synthesis is the process in which ribosomes read genetic instructions in a messenger RNA (mRNA) and assemble a chain of amino acids. Translation in eukaryotes comprises four main phases: initiation, elongation, termination, and recycling (Jackson et al., 2010; Aitken and Lorsch, 2012; Hinnebusch, 2014). The initiation process involves scanning of mRNA for the optimal initiation site and is hypothesized to be impacted by the length of the open reading frame (ORF) and by the position of an AUG codon. Seminal work from Kozak (1986) established that the consensus sequence GCC**R**CCAUGG (in which **R** is a purine, and AUG is the start codon) is the preferred nucleotide sequence for translation initiation. The longest ORF of a transcript is typically considered the only protein coding sequence (CDS) and codes for the reference protein (RefProt) (Furuno et al., 2003). As such, a consensus exists that the majority of eukaryotic mRNAs are monocistronic, implying translation of one distinct protein sequence per transcript. An additional arbitrary criterion for annotating protein-coding ORFs is that transcripts derived from pseudogenes, and transcripts that do not contain an ORF of >100 codons are automatically considered non-coding. The advent of ribosome sequencing (Ribo-seq), a technique that identifies ORFs undergoing translation, shed light on the shortcomings of previous annotations since a large fraction of translated ORFs do not correspond to known CDSs but to unannotated ORFs (Ingolia, 2014). In fact, large-scale Ribo-seq studies and proteogenomics studies including alternative proteins (AltProts) coded by unannotated ORFs identified novel proteins dubbed AltProts (Menschaert et al., 2013; Vanderperre et al., 2013; Ji et al., 2015; Chen et al., 2020). These proteins are sometimes also termed microproteins when they are shorter than 100 amino acids. Here, we will use the term AltProt independently of the length of the unannotated protein. AltProts are translated from regions previously annotated as non-coding, i.e., 5′- or 3′-UTR of mature mRNAs, from ORFs that overlap the CDS in a different reading frame, or from transcripts that were labeled as non-coding. Hence, AltProts are novel proteins and should not be confounded with protein isoforms (Figure 1). Some of these novel proteins fulfill key (patho) physiological functions and thus expand the functional proteome landscape in human cells (Gagnon et al., 2021).

In this review, we analyze and explore AltProts in neurobiology. To begin, we discuss the alternative proteome and briefly review transcription and translation in the brain. Then we discuss the potential mechanisms elemental to aberrant translation initiation and the impact of AltProts on protein diversity. Finally, we review identified AltProts in the brain and discuss their potential role in pathological or healthy neuromolecular processes.

## The alternative proteome

In the past 20 years, multiple mRNAs were found to not only code for a RefProt but also for a novel distinct protein sequence (dual-coding mRNA) (Klemke et al., 2001; Kondo et al., 2007; Autio et al., 2008; Akimoto et al., 2013; Chalick et al., 2016; Delcourt et al., 2017; Samandi et al., 2017). This led to the identification of novel functional proteins (e.g., AltMRVI1, AltSLC35A4, AltCDKN2, and PEP7) (Ouelle et al., 1995; Vanderperre et al., 2013; Andreev et al., 2015; Yosten et al., 2016). These unexpected translation events were considered exceptional and the polycistronic features of mRNA were disregarded in proteomics and functional genomics studies. As such, functional studies of a specific gene are focused on the protein encoded by the reference CDS, or by their isoforms expressed from variably spliced transcripts. Similarly, large-scale mass spectrometric-based proteomics methods rely on conventional annotations that do not include AltProts, precluding their discovery. Indeed, protein identification involves the digestion of proteins followed by the detection of peptide fragments and their corresponding experimental spectra. Subsequently, the data is compared to theoretical spectra that are generated *in silico*, based on protein sequence databases. Identified peptides are then matched to their corresponding proteins. Hence, “identification” of proteins in a biological sample means uncovering the presence of previously annotated proteins rather than identifying the entire set of proteins in the sample. The most popular protein sequence databases are those from UniProtKB (The UniProt Consortium, 2017, 2021). As indicated above, proteins translated from alternative ORFs (AltORFs) are not included in these databases and cannot be detected.

**FIGURE 2:**
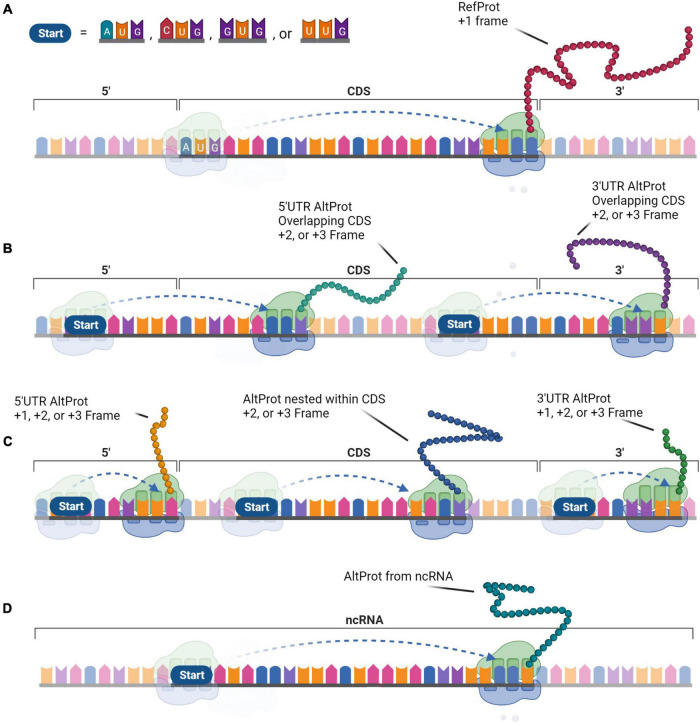
The generation of protein diversity at the translational level. In contrast to the monocistronic transcript paradigm, protein diversity arises from the multi-coding properties of messenger RNA (mRNA) that expand the functional proteome. **(A)** The Reference protein (RefProt) is translated from the coding sequence (CDS) that is initiated by an AUG codon and is enclosed by the 3′- and 5′-UTR. Alternative proteins (AltProts) are translated from unannotated open reading frame (ORFs) that can initiate at AUG, CUG, GUG, or UUG codons. **(B)** These ORFs can overlap the CDS which will generate distinct protein sequences when the ORFs are out-of-frame. **(C)** In addition, translation from ORFs located within the 3′- or 5′-UTR, or from an out-of-frame ORF nested within the CDS will generate distinct protein sequences. **(D)** Finally, AltProts can be translated from transcripts that were previously considered non-coding. This perception can significantly expand protein diversity within cells.

A more comprehensive approach was required to test the hypothesis of the widespread existence of AltProts. Ribosome profiling is the first technique that enables the discovery of ORFs being translated (Brar and Weissman, 2015). This approach sequences ribosome-protected mRNA fragments and thus reveals ribosome positions on these transcripts. As such, Ribo-seq can establish deep insight into global protein translation. However, ribosome occupancy alone is not sufficient to annotate ORFs as a genuine CDS (Guttman et al., 2013). In addition, ORFs overlapping a CDS are difficult to identify with a usual Ribo-seq approach (Bazzini et al., 2014; Smith et al., 2014). To overcome these shortcomings, strategies were developed to stall the initiating ribosomes at the start codon and sequence the protected RNA fragment at the initiation site (Ingolia et al., 2011; Gao et al., 2015). Overall, Ribo-seq led to the detection of thousands of translated AltORF and protein isoforms, in addition to annotated CDSs (Ji et al., 2015; Chen et al., 2020). Isoforms are shorter or longer versions of any RefProt following initiation at an in-frame downstream or upstream initiation codon. AltProts on the other hand, have distinct amino acid sequences that do not share significant similarities with the RefProt translated from the same transcript. The stoichiometry between proteins translated from the same mRNA is largely unknown, but some AltProts were determined to be the main gene product (Delcourt et al., 2018). Translation of these novel proteins can initiate at near-cognate (non-AUG) codons, further expanding the repertoire of prospective protein-coding-ORFs (Figure 2; Chen et al., 2020). The capacity of ORFs starting with near-cognate start codons to produce functional proteins has been demonstrated earlier (e.g., MYC) (Hann et al., 1988).

Of note, the majority of AltProts are shorter than 100 amino acids. In general, small proteins, or peptides, are assumed to not be able to fold into stable structures. As such, they are generally considered non-functional and are therefore excluded from proteome annotations. Yet, small proteins can fulfill key physiological functions (Couso and Patraquim, 2017). Multiple functional endogenous peptides have been detected in the human body (Pauli et al., 2014; Huang et al., 2017; Lin et al., 2019). Of particular note, functional peptides are enriched in the brain. The 38–42 amino acid long Amyloid-beta peptides are involved in the pathogenesis of Alzheimer’s disease, but also display physiological functions, including repairing leaks in the blood-brain barrier (Brothers et al., 2018). The 33 amino acid long peptide Orexin-A is a functional peptide involved in sleep and wakefulness and has recently been linked to dysfunction of hippocampal neurogenesis (Lin et al., 1999; Forte et al., 2021). In fact, neuropeptides are that enriched in the brain that they are appreciated as a specific class (Burbach, 2010). These peptides are generated by proteolytically processing of preproproteins and should therefore not be confounded with AltProts, which are translated directly from mRNA. The contribution of neuropeptides to the functional neuroproteome is a substantial precedent for further exploring small proteins and their function in the brain.

## The convoluted network of transcription and translation in neurobiology

The human brain is an extraordinarily intricate organ with neurons that are connected into vast networks by synapses. At the genomic level, the brain is characterized by an unusually high level of alternative splicing compared to other tissues in the body. Alternative splicing is the process in which pre-mRNA derived from a multi-exonic gene, produces variably spliced transcripts. The expression of these isoforms is restricted to certain tissues and cell types (Shalek et al., 2013). The transcriptome of the human brain exhibits a high number of splicing variants, which significantly increases the transcriptomic and proteomic diversity encoded by the same gene (Yeo et al., 2004; Melé et al., 2015). In approximately half of the alternative splicing events the reading frame is shifted, but these transcripts are often subjected to nonsense-mediated decay due to the presence of a premature termination codon (Lewis et al., 2003; Zhang et al., 2007). As such, the majority of translated isoforms mainly exhibit partial common amino acid sequences. Yet, they can have vastly different interaction profiles and/or an altered subcellular localization (Yang et al., 2016; Yang and Carstens, 2017).

Another specific feature in the brain is found at the translational level: there is no correlation between mRNA levels and the concentration of the corresponding proteins, with the exception of protein classes involved in protein modification, regulation of metabolic and synaptic activity, and proteins associated with a cellular membrane (Liu et al., 2016; Bauernfeind and Babbitt, 2017). The fact that factors beyond mRNA determine protein concentration suggests a prominent role for the regulation of protein translation in the brain. A recent study established that the natural anti-sense transcript, MAPT Antisense RNA 1 (MAPT-AS1), regulates and represses tau production within brain cells (Simone et al., 2021). Silencing MAPT-AS1 led to increased tau levels in neurons and was found to correlate with tau pathologies. The mammalian-wide interspersed repeat, embedded in the anti-sense transcript, competes for ribosomal pairing with the internal ribosomal entry site of MAPT, and thus blocks the translation of tau. The same study reports the involvement of other natural anti-sense transcripts in the regulation of Amyloid-beta precursor protein (APP), which is associated with Alzheimer’s disease, and α-synuclein which is associated with Parkinson’s disease and Lewy body dementia (Spillantini et al., 1997; Jellinger, 2018; Hampel et al., 2021). This finding shows how the translation of proteins involved in neurological disorders can potentially be controlled through the regulation of the interaction between the mRNA and the ribosome.

## Alternative modes of translation initiation

**FIGURE 3:**
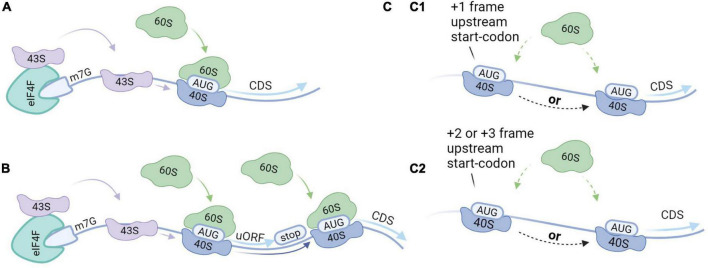
Translation initiation modes. **(A)** Canonical cap-dependent translation; the 43S ribosomal subunit is recruited by the 5′ end of an messenger RNA (mRNA), and subsequently binds to the mRNA and scans for the AUG start codon. The ribosome is dissociated after the translation of the protein. **(B)** Translation re-initiation follows the same steps as a cap-dependent translation but after translation of the protein, the 40S ribosomal subunit stays associated with the mRNA. While moving in the 3′ direction, the 40S ribosomal subunit can bind initiation factors recovering its initiation potential. As such, translation initiation can take place at a downstream start codon. **(C)** Leaky scanning; 43S ribosomal subunits can skip potential start codons when they are located in a weak Kozak sequence. (C1) When the start codons are in the same reading frame and there is no stop codon in between, this can result in the translation of two isoforms from the same transcript. Alternatively, when there is a stop codon in between this will result in the translation of two distinct proteins from the same transcript. (C2) When the start codons are out-of-frame two distinct proteins will be translated from the same transcript.

Reference proteins are generally translated by canonical cap-dependent translation, but the recent discovery of AltProts suggests alternative modes of translation. We provide a brief overview of initiation modes that might be elemental to the translation of AltProts (Figure 3). Non-canonical translation is often linked to survival mechanisms as a response to cellular stress (Bertram et al., 2008; Kwan and Thompson, 2019; Bohlen et al., 2020). Alternative modes of translation are therefore often linked to cancer (Sriram et al., 2018). We believe that cellular stresses may induce AltProt translation in pathologies or disturb the stoichiometry between AltProt and RefProt translation from a specific transcript. Therefore, AltProts might play a role in diseases in which oxidative stress is involved in their pathogenesis such as several types of dementia (Butterfield and Halliwell, 2019).

### Canonical cap-dependent translation

Reference proteins are generally translated by a mechanism that is dependent on both the 7-methylguanosine cap (m7G), which is located at the 5′ end of an mRNA, and ribosome scanning (Aitken and Lorsch, 2012). The process starts when transfer-RNA (tRNA) and Guanosine-5′-triphosphate (GTP) bind to the eukaryotic initiation factor 2 (eIF2) complex, yielding a ternary complex that assembles with the small ribosomal subunit (40S) (Kimball, 1999). Together with other initiation factors the 40S forms the pre-initiation complex (43S). The 43S complex is then recruited to the 5′ end of an mRNA marked with the m7G cap and the eukaryotic initiation factor 4F (eIF4F) complex among other factors (Fraser, 2015). The 43S complex subsequently scans the 5′-UTR for a start codon within the correct nucleotide sequence. Scanning stops at the start codon and the initiation factors are released. The large ribosomal subunit (60S) then binds to the 40S complex on the mRNA to form the 80S ribosome to enable translation elongation.

### Leaky scanning

Leaky scanning can occur when multiple potential start codons are present on the transcript and the first start codon is not in a strong Kozak context (Wang and Rothnagel, 2004; Palam et al., 2011). As such, the 43S complex may initiate at the first start codon, or skip the first start codon, continue scanning and initiate at a downstream start codon. If the start codons are in the same reading frame and there is no stop codon in-between, two protein isoforms are produced. Alternatively, if there is a stop codon in-between the start codons, or if the start codons are out-of-frame, this will result in distinct proteins. The frequency of leaky scanning therefore determines the stoichiometry between the RefProt and the AltProt or between isoforms that are translated from the same mRNA.

### Translation re-initiation

Approximately half of all human mRNA contains at least one small ORF upstream of the CDS, also termed an upstream ORF (uORF) (Calvo et al., 2009). Translation re-initiation can occur in transcripts that exhibit start codons in a strong Kozak sequence. The initiation starts at the uORF and the protein is translated. After translation of the uORF, the 40S complex can remain associated with the mRNA. While moving in the 3′ direction, the 40S subunit can recover initiation capacity by binding the initiation factors. In the absence of the initiation factors, the 40S complex may skip subsequent start codons. As such, translation re-initiation results in the translation from downstream initiations sites.

## Alternative proteins and the regulation of reference protein translation

Upstream open reading frames (uORFs) were initially thought to exclusively act as negative regulators for the translation of the RefProt. In this mechanism, the translation of the RefProt is inhibited because the ribosome is dissociated into the small and the large subunit before reaching the CDS. Alternatively, ribosomes can be stalled at the uORF and therefore block the translation of subsequent ORFs (Young et al., 2016). In both translation initiation modes, leaky scanning and re-initiation, proteins can be translated from the 5′-UTR. Recently, a large-scale study established that disruption of upstream translation is associated with human disease (Lee et al., 2021). Thus far, it is not clear if translated uORFs that regulate RefProt expression in addition exhibit the capacity to produce functional AltProts, or if they should be considered as a separate subset of proteins.

## Somatic mutation as a possible mechanism for *de novo* generation of protein-coding sequences

**FIGURE 4:**
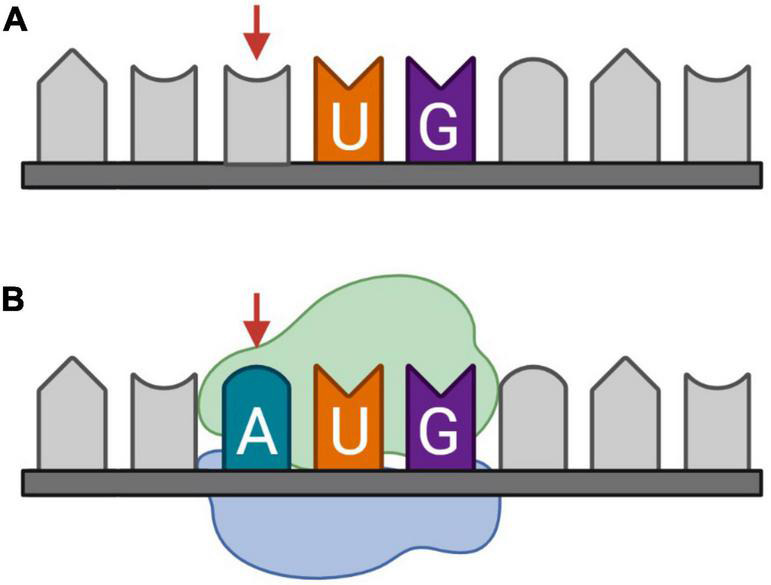
Somatic mutation, more specifically synonymous single-nucleotide variants (SNVs), as a candidate mechanism for *de novo* generation of protein-coding sequences (CDS). **(A)** Messenger RNA (mRNA) transcribed from the reference DNA, **(B)** mRNA transcribed from DNA with an SNV on the third nucleotide. The introduction of an adenine before uracil and guanine, respectively, forms an AUG codon on which initiation potentially takes place.

Somatic mutations are variations in the DNA that are acquired after conception (Biesecker and Spinner, 2013). These postzygotic variants can generate genetically distinct cells within a single organism and are known to appear and accumulate in the brain during development and aging. This might be due to errors in DNA replication, or defects in DNA repair mechanisms induced by extensive oxidative stress (Wang et al., 2014; Maynard et al., 2015). The accumulation of somatic mutations in neurons during aging, or genosenium, has been linked to dysregulation of tau phosphorylation and Alzheimer’s disease (Park et al., 2019; Miller et al., 2021). The mutations include synonymous and non-synonymous single-nucleotide variants (SNVs). Synonymous SNVs are in general disregarded because they do not change the amino acid sequence of the protein. However, synonymous variations in the reading frame of the CDS may be non-synonymous and have a great impact on an overlapping AltORF (Brunet et al., 2021a). Furthermore, variations of low significance within untranslated regions (UTRs) may have a significant impact if they result in a missense mutation within AltORFs. Finally, variations may create translation initiation sites, resulting in a functional ORF coding a novel AltProt (Figure 4).

## Impact of alternative proteins on proteomic- and functional diversity

The field of functional genomics uses genomics, transcriptomics, proteomics, and interactomics analyses to better understand the genotype to phenotype relationship. The discovery of unannotated ORFs adds another level of complexity. OpenProt is a proteogenomic resource including AltProts and novel isoforms in addition to RefProts. This resource uses a comprehensive prediction method based on a 3-frame *in silico* translation of transcripts retrieved from Ensembl and NCBI RefSeq. The output yields an exhaustive database of predicted proteins coded by any ORF with a minimum size of 30 codons in the transcriptome of 10 species. In the latest release (OpenProt v1.6), OpenProt also includes the initiation of AltProts at non-AUG codons (Brunet et al., 2021b). Based on the re-analyses of mass spectrometry-based proteomics and ribosome profiling data, OpenProt provides expression evidence for more than 40,000 AltProts in humans, largely expanding the proteome listed in conventional databases. As more studies use the OpenProt annotation to discover new proteins, it is expected that the number of AltProts will increase accordingly. OpenProt does not annotate microproteins smaller than 30 amino acids. As such, very small peptides like PEP7 would remain undetected using OpenProt databases (Yosten et al., 2016). Hence, determining the contribution of very small proteins to the proteome is still a challenge. Elucidating the function of thousands of AltProts is not an easy endeavor. Investigations on small proteins comes also with significant technological challenges. Despite these challenges, we predict that including AltProts in proteomics studies will likely help discover biomarkers for precision medicine.

## Exploring the alternative neuroproteome

Alternative proteins elude detection in the brain because they are not included in protein databases and the potential dual-coding feature of mRNAs and coding potential of RNAs annotated as non-coding are overlooked. However, there are some substantial arguments to investigate the existence of these novel neuroproteins. First, weak Kozak sequences are enriched in genes involved in neurobiology, which might lead to increased leaky scanning and downstream initiation events (Acevedo et al., 2018). Second, approximately 40% of long non-coding RNAs (lncRNAs) are specifically expressed in the brain, which increases the potential translation of AltProts from ncRNA (Derrien et al., 2012). Finally, somatic mutations can potentially create a translation initiation site for a new AltORF. As such, AltProts might be translated in age-related neurodegenerative disorders. Although no proteome discovery study specifically targeted the alternative neuroproteome, multiple AltProts have been detected in the brain. Humanin is translated from a short ORF from mitochondrial ribosomal RNA (mt rRNA). It was detected serendipitously in surviving brain cells of familial Alzheimer’s patients and was found to protect against Amyloid-beta toxicity (Hashimoto et al., 2001a,b; Tajima et al., 2002). In addition, it is involved in multiple neuroprotective processes such as inhibition of inflammatory responses, protection against neuronal cell death, and prevention of synapse loss (Sponne et al., 2004; Zhao et al., 2013; Zárate et al., 2019). Due to its cytoprotective properties it is now considered a therapeutic target for degenerative diseases (Zuccato et al., 2019).

Several studies identified AltProts in the brain encoded in dual-coding transcripts. For instance, the alternative prion protein (AltPrP) is translated from an out-of-frame ORF (+3 reading frame) nested within the annotated CDS of the prion protein (PRNP) locus (Vanderperre et al., 2011). The RefProt, the PrP is involved in the pathogenesis of transmissible spongiform encephalopathies (TSEs). The function of AltPrP is unknown, but the increased expression after proteasome inhibition and endoplasmic reticulum stress might indicate its biomarker potential in TSEs or other neurodegenerative diseases like Parkinson’s and Alzheimer’s (Yoshida, 2007; Salminen et al., 2009; Vanderperre et al., 2011). The transcript coding for the A2A Adenosine receptor (A2AR) is also dual-coding (Lee et al., 2014). A2AR is a drug target for multiple neurodegenerative disorders like Parkinson’s and Huntington’s (Chiang et al., 2009; Kulisevsky and Poyurovsky, 2012). The AltProt AltA2AR is translated from an out-of-frame ORF (+2 reading frame) that starts in the 5′-UTR and partially overlaps the CDS. Stimulation of A2AR increased translation of the AltProt, and upregulation of A2AR transcripts led to increased translation of both the RefProt and the AltProt. AltA2AR was found to be involved in the modulation of the expression of several genes in the mitogen-activated protein kinase (MAPK) pathway. The AltProt AltAtaxin-1 is expressed from the same mRNA as Ataxin-1 but is translated from an out-of-frame (+3 reading frame) ORF that is nested within the CDS (Bergeron et al., 2013). Ataxin-1 is involved in the neurodegenerative disorder spinocerebellar ataxia type 1 and is moreover associated with increased Alzheimer’s disease risk (Orr et al., 1993; Bertram et al., 2008; Suh et al., 2019). The AltProt is expressed in normal and in pathological conditions but whether it has a function in spinocerebellar ataxia type 1 is unknown. More recently, a transcript from the FUS gene was found to be bicistronic. AltFUS is translated from an out-of-frame ORF nested within the CDS (Brunet et al., 2021a). The RefProt FUS has been linked to the neurodegenerative diseases amyotrophic lateral sclerosis (ALS) and frontotemporal dementia (Deng et al., 2014; Nolan et al., 2016). AltFUS was found to induce motor neuron toxicity. Thus, the RefProt and AltProt contribute both to FUS-mediated toxicity (Brunet et al., 2021a). Finally, the presence of AltProts was demonstrated in extracellular vesicles (EVs) produced by glioma cells that can contribute to carcinogenesis (Murgoci et al., 2020). In total, six AltProts were identified among other proteins involved in tumor progression. This study not only highlights the usefulness of targeting glioma EVs for diagnosis but also indicates the biomarker potential for AltProts.

## Perspective

Ribosome sequencing studies shifted the paradigm of RNA expression in eukaryotes by identifying novel translated ORFs in regions of transcripts previously believed to be non-coding. Some of these novel proteins are stable, bioactive, and/or functionally independent of the RefProt translated from the same transcript. Yet, conventional protein sequence databases do not include AltProts because their role is not well understood, which in turn hampers subsequent research for determining their function.

**FIGURE 5:**
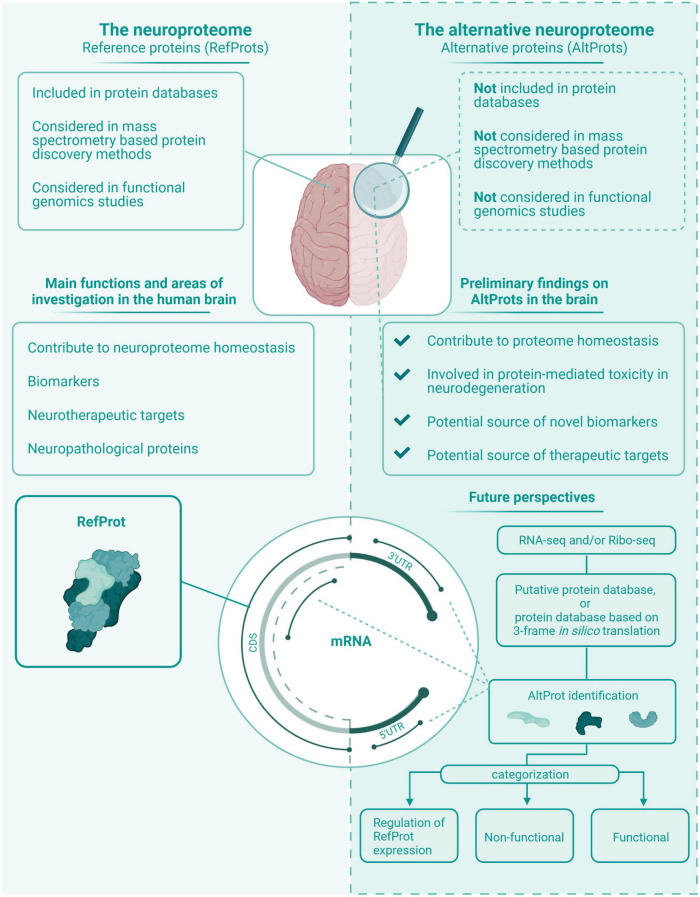
The neuroproteome vs. the alternative neuroproteome. This value proposition of the alternative neuroproteome highlights the key findings in this exploratory review article. The alternative neuroproteome is an undiscovered area that can increase our understanding of the intricate neuroproteome and is a novel source of potential biomarkers and therapeutic targets. However, subsequent studies have to determine which alternative open reading frame (ORFs) are protein coding, and if the translated alternative protein (AltProt) is either functional, non-functional, and/or involved in the regulation of reference protein (RefProt) translation.

The neurotranscriptome is characterized by a large fraction of canonical translation initiation sites with a weak Kozak sequence and a significant number of lncRNA. These two features suggest that neuronal AltORFs are numerous and their translation may be increased. Ribo-seq on the neurotranscriptome and proteomics studies on the neuroproteome would help determine the extent of the translation of AltORFs. Non-canonical translation events are linked to cellular stress responses, which might indicate their implication in carcinogenesis and neurodegenerative diseases like Alzheimer’s, Parkinson’s, TSEs, and ALS. There are several examples in the literature of functional AltProts in the brain with functions associated with normal or pathological states. This novel group of proteins can therefore be important to help unravel (patho)physiological functions, and potentially represents novel biomarkers in neurodegenerative diseases and therapeutic targets. The field of AltORFs and AltProts is recent, and much work needs to be done to determine the role of AltProts in the nervous system (Figure 5). As such, efforts to advance research on non-canonical ORF will likely advance the field (Mudge et al., 2021).

Very large databases, including those provided by OpenProt are useful for the discovery of AltProts but they are too large and cannot be routinely used with typical proteomic workflows. OpenProt allows subdivision by species, genes, transcripts, proteins, ncRNAs, mRNAs, or by the locations on mRNA (e.g., 5′-UTR, CDS, etc.). Additionally, it is possible to exclusively select AltProts with a minimum degree of experimental evidence. Currently, it is not possible to further divide ncRNA by biotype. Studies specifically targeting a specific biotype (e.g., processed pseudogenes) could leverage this additional filter, allowing usage of a restricted database. For example, ncRNA can be subdivided into short ncRNA, long ncRNA, and pseudogenes. Alternatively, a more comprehensive subdivision by biotype could be achieved by incorporating the biotype annotation from Ensembl/Havana or Gencode (Aken et al., 2016; Frankish et al., 2021). However, the most comprehensive approach for deciphering a cellular proteome would be to generate custom databases with RNA-sequencing data. In this approach, the protein sequence database is built from ORFs present in transcripts identified by RNA-seq rather than built with all predicted ORFs in the genome. In addition to providing a smaller database, RNA-seq data enables the identification of genetic variants.

The discrepancy between conventional annotations and experimental translational landscapes stresses the necessity for a better understanding of the mechanism of translation initiation and its regulation. In particular, transcripts may encode several proteins rather than a single protein as predicted from conventional annotations. A strong Kozak sequence is certainly an important feature for maximal translation of a specific ORF, yet a significant fraction of initiation sites, particularly in genes involved in neurobiology does not fit a consensus Kozak sequence.

## Concluding remarks

The detection of novel functional ORFs is essential to further unravel the neuroproteome. The purpose of this review was to create awareness that assumptions in genome annotations have created an unintentional bias in proteomics research. Particularly in a convoluted environment like the human brain, it is important to consider the multi-coding potential of transcripts to relate gene expression and function in physiological and pathological settings. The identification and mapping of AltORFs and functional AltProts are elemental for widespread acceptance of the potential multi-coding properties of eukaryotic mRNA and to contradict the “non-coding” theorem.

## Author contributions

PM, CH, and SL conceptually designed the work. PM, XR, CH, CD, and JV wrote the manuscript. PM made the illustrations. CH, XR, and SL reviewed the manuscript. All authors read, commented on the manuscript, and approved the final version.
